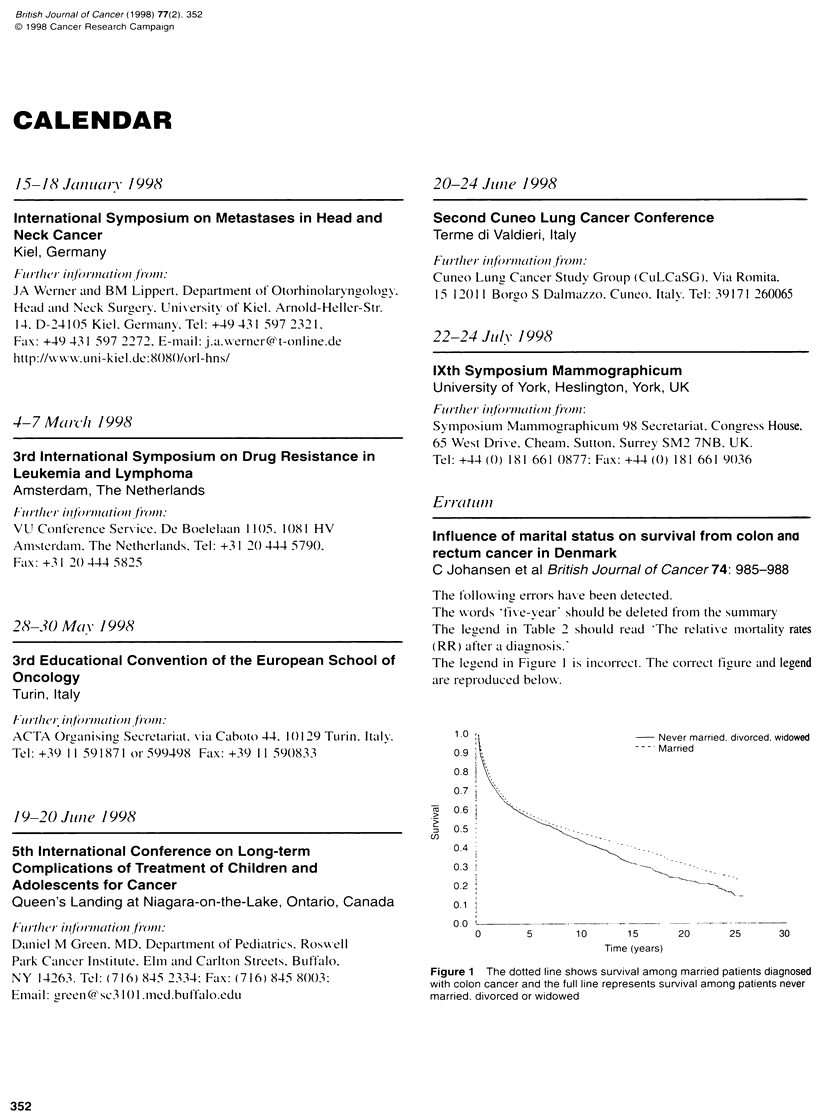# Calendar

**Published:** 1998

**Authors:** 


*British Journal of Cancer* (1998) **77**(2). 352
© 1998 Cancer Research Campaign

### CALENDAR

### *15–18 January 1998*

**International Symposium on Metastases in Head and Neck Cancer**
Kiel, Germany

*Further information from:*

JA Werner and BM Lippert, Department of Otorhinolaryngology, Head and Neck Surgery, University of Kiel, Arnold-Heller-Str. 14, D-24105 Kiel, Germany, Tel: +49 431 597 2321, Fax: +49 431 597 2272, E-mail: j.a.werner@t-online.de http://www.uni-kiel.de:8080/orl-hns/

### *4–7 March 1998*

**3rd International Symposium on Drug Resistance in Leukemia and Lymphoma**
Amsterdam, The Netherlands

*Further information from:*

VU Conference Service, De Boelelaan 1105, 1081 HV Amsterdam, The Netherlands, Tel: +31 20 444 5790, Fax: +31 20 444 5825

### *28–30 May 1998*

**3rd Educational Convention of the European School of Oncology**
Turin, Italy

*Further information from:*

ACTA Organising Secretariat, via Caboto 44, 10129 Turin, Italy. Tel: +39 11 591871 or 599498 Fax: +39 11 590833

### *19–20 June 1998*

**5th International Conference on Long-term Complications of Treatment of Children and Adolescents for Cancer**
Queen's Landing at Niagara-on-the-Lake, Ontario, Canada

*Further information from:*

Daniel M Green, MD, Department of Pediatrics, Roswell Park Cancer Institute, Elm and Carlton Streets, Buffalo, NY 14263. Tel: (716) 845 2334; Fax: (716) 845 8003; Email: green@sc3101.med.buffalo.edu

### *20–24 June 1998*

**Second Cuneo Lung Cancer Conference**
Terme di Valdieri, Italy

*Further information from:*

Cuneo Lung Cancer Study Group (CuLCaSG), Via Romita, 15 12011 Borgo S Dalmazzo, Cuneo, Italy. Tel: 39171 260065

### *22–24 July 1998*

**IXth Symposium Mammographicum**
University of York, Heslington, York, UK

*Further information from:*
Symposium Mammographicum 98 Secretariat, Congress House, 65 West Drive, Cheam, Sutton, Surrey SM2 7NB, UK. Tel: +44 (0) 181 661 0877; Fax: +44 (0) 181 661 9036